# Correcting Classifiers for Sample Selection Bias in Two-Phase Case-Control Studies

**DOI:** 10.1155/2017/7847531

**Published:** 2017-09-24

**Authors:** Norbert Krautenbacher, Fabian J. Theis, Christiane Fuchs

**Affiliations:** ^1^Institute of Computational Biology, Helmholtz Zentrum München, German Research Center for Environmental Health, Munich, Germany; ^2^Department of Mathematics, Technische Universität München, Munich, Germany

## Abstract

Epidemiological studies often utilize stratified data in which rare outcomes or exposures are artificially enriched. This design can increase precision in association tests but distorts predictions when applying classifiers on nonstratified data. Several methods correct for this so-called sample selection bias, but their performance remains unclear especially for machine learning classifiers. With an emphasis on two-phase case-control studies, we aim to assess which corrections to perform in which setting and to obtain methods suitable for machine learning techniques, especially the random forest. We propose two new resampling-based methods to resemble the original data and covariance structure: stochastic inverse-probability oversampling and parametric inverse-probability bagging. We compare all techniques for the random forest and other classifiers, both theoretically and on simulated and real data. Empirical results show that the random forest profits from only the parametric inverse-probability bagging proposed by us. For other classifiers, correction is mostly advantageous, and methods perform uniformly. We discuss consequences of inappropriate distribution assumptions and reason for different behaviors between the random forest and other classifiers. In conclusion, we provide guidance for choosing correction methods when training classifiers on biased samples. For random forests, our method outperforms state-of-the-art procedures if distribution assumptions are roughly fulfilled. We provide our implementation in the R package* sambia*.

## 1. Introduction

Statistics is an art of inferring information about large populations from comparably small random samples. This is necessary because in practice it is most often impossible to receive measurements from all individuals in a population (e.g., due to organizational or cost reasons). In the clinical context, for example, one might aim to predict the risk for a certain disease based on clinical features for an entire population. The risk model will be derived from information from a much smaller random subsample of the population. When building such models, a common assumption is that the subsample follows the same distribution as the population the sample was taken from. This assumption, however, is not valid if the sample is not taken at random. In the epidemiological context, for example, this case occurs in the well-known* case-control studies* [1]. Here, one is interested in finding associations between features and rare disease outcomes. In order to increase precision and achieve higher statistical power for finding significant associations, cases are enriched such that cases and controls are equally represented in the sample. When a case-control study is used for risk prediction on an unbiased population (e.g., via logistic regression), certain adjustments have to be made which have been elaborated in [2–5].

**Figure 1:**
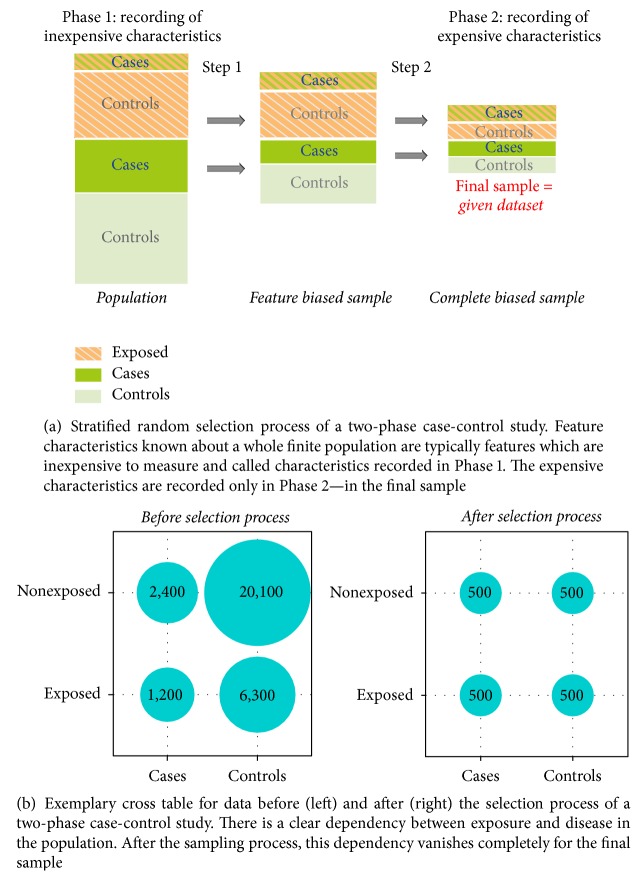


An even more complex sample design appears in* two-phase case-control studies* [6, 7]. Here, one enriches not only a rare disease outcome but also a rare covariate (e.g., an exposure). This measure prevents the sample from containing only few individuals that fall into both rare categories. From such a sample, one would hardly be able to draw conclusions about the rare combination.  Figure 1(a) illustrates how the sampling procedure is performed in practice.  Figure 1(b) shows an exemplary table of numbers of cases/controls and exposed/nonexposed individuals in the population and the sample. This and other complex survey designs (e.g., cohort sampling designs [8]) have been used in order to obtain subpopulations with rare characteristics of features of interest [9–11]. The efficiency and analysis of the design are described in [6].

In the situations described above, the sample follows a different distribution than the population. This can affect statistical analysis. In the general context, this issue is known as* sample selection bias* [12–14]. It generally occurs when not all individuals from the population have the same probability of getting selected for the sample. If a statistical estimate is affected by sample selection bias, one should correct for it. The question of whether correction is necessary depends on the type of sample selection bias, the considered classifier, and the research question to be answered. For example, no adjustment is required if only the outcome variable is enriched and logistic regression is applied for prediction purposes, because the slope coefficients of the linear predictor remain asymptotically unaffected by sample selection bias for this case (if the functional form and the explanatory features for the model are correct) [15]. In general, however, correction is required, and there are several solutions to encounter this problem in complex survey designs [16, 17]. These existing approaches mainly focus on classical prediction methods or simple survey designs. Strategies applicable also for machine learning approaches have been suggested in the general sample selection bias context [12, 18, 19]. These methods reconstruct the population data or its covariance structure and typically involve nonparametric resampling techniques like bootstrapping. However, they neglect complex survey designs. Thus, while correcting for sample selection bias in logistic regression is well investigated, its consideration is unclear for most machine learning approaches.

**Figure 2:**
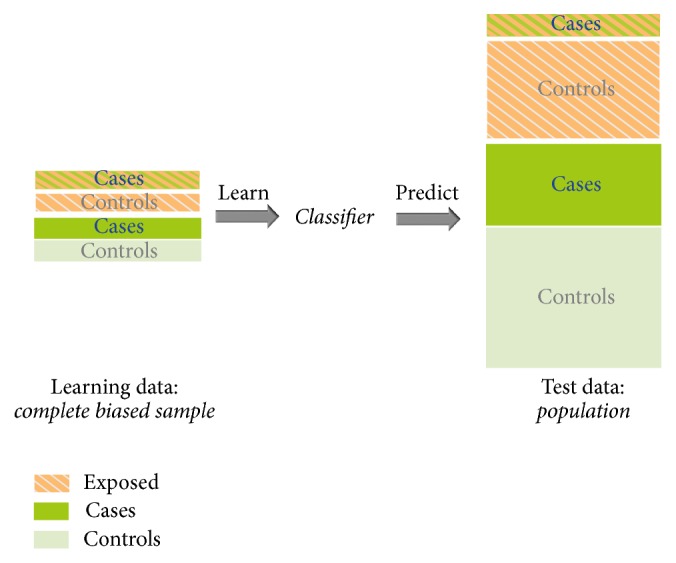
Scheme of learning on biased learning data and predicting on unbiased test data. The classifier learns on four equally sized strata (complete biased learning data set) but predicts on a data set (unbiased population) of different sizes of the four strata.

This paper assesses, proposes, and compares approaches to correct for sample selection bias in complex surveys, especially in two-phase case-control studies. Therefore, we focus on the binary outcome.  Figure 2 illustrates the issue to be addressed. The emphasis is on a widely used machine learning approach: the random forest. We correct for the covariance structure of the sample by incorporating knowledge about the sample selection procedure into nonparametric and parametric resampling techniques. As the random forest is based on resampling anyway (in terms of bagging; see Section 3.2), we incorporate the correction step into the inherent resampling procedure. We compare our correction approaches to analogous state-of-the-art approaches, both for the random forest and for other common classifiers, namely, logistic regression, logistic regression including interaction terms, and the naive Bayes classifier. We especially address the question of whether correction is necessary in random forests, and if so, whether current correction approaches can successfully be transferred to the random forest and whether improvement is possible through alternative approaches. We assess and compare the prediction performance of the correction techniques in a synthetic simulation study and in a real data application. We provide the R package* sambia* so that readers can easily apply the methods presented here to their data.

This paper is structured as follows. We formalize sample selection bias and address the necessity of correction in Section 2.  Section 3 explains current approaches for corrected learning on biased samples, and we propose two new methods based on drawing observations from theoretical distributions assumed for the given data. We furthermore analyze properties of the various approaches in the context of sample selection bias.  Section 4 presents a simulation study which compares all approaches regarding performance on new unbiased test data.  Section 5 shows a similar analysis on real data. We discuss and conclude our work in Section 6.

## 2. Preliminaries

This section introduces general definitions and background information: a formal description of sample selection bias (Section 2.1), the special case of two-phase case-control studies (Section 2.2), and properties of biased samples (Section 2.3).

### 2.1. Sample Selection Bias

The following setup is similar to that of Zadrozny [12] and distinguishes* sample selection bias* into three types. We assume a set of observations {(**x**_*i*_, *y*_*i*_)}_*i*=1,…,*n*_ which are drawn independently from a distribution *D*. The domain of *D* is *𝒳* × *𝒴* with *𝒳* being the feature space and *𝒴* being a measurable space. Here, *𝒴* is a discrete binary label space since we focus on binary classifiers in this work. Throughout the paper, we will denote random variables by capital letters and realizations (i.e., observations in the sample) by lowercased letters.

For the setup of the sample selection bias issue, let in addition *𝒮* be a binary space. *S* ∈ *𝒮* is the variable that controls the selection of observations: For *s*_*i*_ = 1, the *i*th observation is selected; for *s*_*i*_ = 0, the observation is not selected. Thus, observations (**x**_*i*_, *y*_*i*_, *s*_*i*_) are drawn from a distribution *ℒ* with domain *𝒳* × *𝒴* × *𝒮*.

In general, a sample {(**x**_*i*_, *y*_*i*_, *s*_*i*_)}_*i*=1,…,*n*_ can be biased in three different ways. These types of sample selection bias can be described as follows [12, 19]:

- Label bias: biasedness depends on *Y* only, so *P*(*S*∣**X**, *Y*) = *P*(*S*∣*Y*) but *P*(*S*∣*Y*) ≠ *P*(*S*).
- Feature bias: biasedness depends on **X** only, so *P*(*S*∣**X**, *Y*) = *P*(*S*∣**X**) but *P*(*S*∣**X**) ≠ *P*(*S*).
- Complete bias: biasedness depends on **X** and *Y*; that is, there is no independence between *S* and **X**, *Y*, so *P*(*S*∣**X**, *Y*) ≠ *P*(*S*∣*Y*) and *P*(*S*∣**X**, *Y*) ≠ *P*(*S*∣**X**).

Under label bias, *S* is not necessarily independent of **X** ([19]; for details, see also Appendix A), and for feature bias *S* is not necessarily independent of *Y*.

Whenever there is sample selection bias, there are* selection probabilities P*(*S* = 1∣*Y*, **X**) (in particular *P*(*S* = 1∣*Y*) for label bias and *P*(*S* = 1∣**X**) for feature bias). In practice, these probabilities can often be estimated if they are unknown. Throughout this paper, we assume them to be provided. All approaches proposed in this paper will incorporate these selection probabilities in terms of weights corresponding to the inverse probabilities *P*(*S* = 1∣**X**, *Y*)^−1^.

### 2.2. Sample Selection Bias in Two-Phase Case-Control Studies

In this paper, we will discuss the special case of two-phase case-control studies and hence put them into the context of sample selection bias in this subsection.

The case-control study is an example for sample selection bias in the clinical context: Some diseases under investigation are very rare in the entire population. A random sample of study participants would contain very few cases of the disease. Statistical analysis would suffer from low precision and thus low power. In order to increase precision and power, the number of cases is enriched such that the proportion of cases and controls in the sample is identical. In particular, *P*(*Y* = 1∣*S*) = 0.5 whereas the prevalence rate *P*(*Y* = 1) is much smaller, so *P*(*Y* = 1∣*S*) ≠ *P*(*Y* = 1). This by Bayes' theorem implies *P*(*S*∣*Y* = 1) ≠ *P*(*S*), and thus there is label bias.

Case-control studies are mostly used for investigating associations between disease and features. The underlying label bias does not alter the effect estimates in hypothesis testing for associations between disease and features. However, this is true only asymptotically, and there may be consequences in small sample scenarios. If one focuses on prediction, for example, via logistic regression, as we do in this paper, the intercept estimate can simply be adjusted as described in Rose and van der Laan [4] or Steyerberg et al. [2]. Elkan [20] offers a solution for arbitrary classifiers.

In* two-phase case-control studies*, on the other hand, the selection is additionally controlled by a categorical feature variable. Such studies suffer from label* and* feature bias, so there is complete bias. We focus on this case (i.e., complex survey designs which involve complete bias).

### 2.3. Stratified Random Samples

When data is sampled as in one-phase or two-phase case-control studies, there are groups within which the selection probabilities are equal. These groups are called* strata*. In this paper, we focus on two-phase case-control studies where the strata are determined by a categorical stratum feature (often an exposure) *X*_*e*_ and the outcome *Y*. The remaining features of **X** are $\tilde{X}≔X\setminus X_{e}$.

For a population of size *N* and sample size *n*, let *h* ∈ {1,…, *H*} be the index of the stratum. Realizations falling into stratum *h* are denoted by ${\tilde{x}}_{h}$, *x*_*e*__*h*_, and *y*_*h*_ or combined as $\left(x_{h},y_{h}\right)=\left({\tilde{x}}_{h},{x_{e}}_{h},y_{h}\right)$. We denote by *n*_*h*_ the size of the stratum *h* in the sample and by *N*_*h*_ its size in the population. Then, clearly, *P*(*S* = 1) = *n*/*N* and

(1) PS=1 ∣ x,y=PS=1 ∣ xe,y=PS=1 ∣ hxe,y=nhxe,yNhxe,y,

where *h*(*x*_*e*_, *y*) denotes the stratum determined by *x*_*e*_ and *y*. Throughout the paper, we will simply abbreviate this by *h*.

If the features determining the selection probabilities are categorical, the data set can be partitioned into corresponding strata with equal selection probabilities. This is not the case if, for example, the feature causing the selection bias is continuous. In the categorical case, selection probabilities can be used for adjusting the distribution of the sample to the original distribution of the population.

Consider the selection probability *P*(*S* = 1∣*h*) for an observation of stratum *h*. We define

(2) $$\begin{array}{c} w_{h}≔\left[\;\frac{\max_{h^{\prime }}P\left(S=1\;|\;h^{\prime }\right)}{P\left(S=1\;|\;h\right)}\;\right] \end{array}$$

as the* inverse-probability (IP) weight* for stratum *h*. The squared brackets denote rounding to the closest integer. The term* IP weight* is sometimes used in the literature for the simple inverse selection probability *P*(*S* = 1∣*h*)^−1^. In this work, we use *w*_*h*_ rather than *P*(*S* = 1∣*h*)^−1^ to keep the number of newly generated observations minimal.

In our correction approaches, we will use

(3) $$\begin{array}{c} n^{\prime }≔\overset{H}{\underset{h=1}{\sum }}n_{h}w_{h}, \end{array}$$

which can be seen as the number of reweighted observations (i.e., the sum of all observations multiplied by their weights). As stated above, we are interested in adjustment methods which can be applied to arbitrary classifiers. In the next section, after stating a typical setup of a statistical learning procedure, we will describe several sample selection bias correction approaches proposed in the literature.

## 3. Methods

In this section, we describe, modify, and analyze IP weight-incorporating classifiers which are designed for learning on an unbiased data set, when only a biased data set for learning is given.

### 3.1. Correction Approaches

All approaches adjust the given data set to correct for sample selection bias by reconstructing the original (unbiased) data structure before or while learning the classifier. We consider the classifier

(4) $$\begin{array}{c} \varphi :\left\{\begin{array}{c} {\left(X\times Y\right)}^{\times n}\times X⟶Y\\ \left(\left(x,y\right),X\right)⟼\varphi \left(\left(x,y\right);X\right), \end{array}\right. \end{array}$$

where the given learning data set (**x**, *y*) = ((**x**_1_, *y*_1_),…, (**x**_*n*_, *y*_*n*_)) is mapped to the prediction (in our case classification) rule and applied to the random variable **X**.

#### 3.1.1. State of the Art

The methods in this section were proposed in the literature and are partly modified for our purposes.

*No Correction.* The naive approach for learning on a biased sample is to simply ignore the bias. No IP weights are used, and the classifier is trained on the given sample as it is. As shown by Zadrozny [12], this approach is valid for some cases of sample selection bias, namely, for feature bias for a specific type of classifiers.

*Inverse-Probability Oversampling.* An intuitive method for correcting for sample selection bias is the plain replication of each observation in the sample according to its IP weight (i.e., in a stratified random sample, one replicates an observation of stratum *h* by the factor *w*_*h*_). Then, the number of observations in the reconstructed sample is *n*′. This sample is used for learning. In maximum likelihood-based approaches like generalized regression models, this method is equal to weighting the single likelihoods per observation. The procedure, sometimes simply called* inverse-probability weighting*, has been used early [21], with applications both in regression [22] and in general statistical learning [20]. We refer to this technique as* IP oversampling*: Since in the stratification process some observations were* oversampled*, this method is a way of reoversampling underrepresented observations in the stratified sample. Since IP oversampling is applicable to arbitrary classifiers, we take it into account for further comparisons. A drawback is that it changes the covariance structure per stratum *h*. In Section 3.1.2, we propose a method that corrects for this issue.

*Inverse-Probability Bagging.* Another correction method uses bootstrap aggregation and averaging, commonly abbreviated to the acronym* bagging*. The procedure averages several predictions trained on an ensemble of bootstrap samples and thus makes learners more robust [23]. Nonparametric bootstrap samples arise by randomly drawing *n* times from the original data set of size *n* with replacement. Bagging procedures fit a learner on each of these bootstrap samples and combine the learners by averaging predictions or by majority vote. When building bootstrap samples from biased data sets, as in our case, resampling can take into account IP weights: Instead of drawing observations randomly, selection probabilities are set proportional to *w*_*h*_ for the respective strata *h*. This procedure is proposed by Nahorniak et al. [24] and labeled as* IP bagging* here.

*Costing.* Zadrozny et al. [18] argue that sampling with replacement as done in IP bagging is inappropriate since sets of independent observations from continuous distributions contain two identical elements only with zero probability, whereas nonparametric bootstrap samples generally contain observations repeatedly. Zadrozny et al. [18] propose an approach called* costing*, which is similar to IP bagging in terms of resampling from the learning data and aggregation of learned algorithms on *m* new samples. It differs in the implementation of resampling the *m* learning sets: Here, an observation from the original learning set enters a resampled data set only once at most. It is selected with probability *w*_*h*_/max_*h*′_*w*_*h*′_ according to the corresponding stratum *h*. Consequently, the size of the new samples is smaller than *n* and generally varies among the *m* learning sets. The latter aspect indicates the difference of this approach to subsampling without replacement. A detailed description of the aspects of the algorithm can be found in Zadrozny et al. [18], Sections  2.3.2 to 2.3.4.

A drawback of costing in case of strata with a low number of observations is the following: There may be subsamples which do not contain observations from all strata, which implies that no classification rule can be learnt for the missing strata from those subsamples. For the purposes of this paper, we adjusted the costing algorithm by not taking into account such incomplete samples. This modification causes bias which we consider negligible.

*Modified SMOTE.* So far, all correction approaches replicated given observations. In contrast, [25] proposed a* synthetic minority oversampling technique (SMOTE)* to generate new, synthetic data. The strategy is designed as a solution for the imbalanced class problem, where rare cases (the* minority class*) are hardly represented in the (nonstratified) sample, which mainly consist of common cases from the* majority class*. In this situation, several classifiers perform poorly because of the imbalanced proportion of outcome categories in the data.

In its original form, SMOTE generates synthetic observations for the minority class as follows: For fixed *k* ∈ *ℕ*, one determines the *k* nearest neighbors of the minority class. Depending on the desired number of new observations, one then randomly selects a corresponding amount of instances from this neighborhood. New observations arise as weighted averages between original feature vectors and selected nearest neighbors. To that end, weights are randomly sampled from the unit interval.

We adapt SMOTE to the context of stratified random samples: Rather than enlarging only the minority class, we generate synthetic observations for all strata with *w*_*h*_ > 1. Thus, we apply SMOTE up to *H* − 1 times, once for each stratum which requires more observations. We refer to this algorithm as* modified SMOTE* hereafter.

#### 3.1.2. Correcting Covariance Structures

The approaches above aim to reconstruct the original data distribution in order to then learn a classifier on an unbiased sample. However, several aspects are not incorporated so far: IP oversampling replicates observations and by this biases the covariance structure within the strata. A correction for this biasedness should be provided. Similarly, modified SMOTE biases the data, especially for large weights *w*_*h*_, where the same observations are used several times for synthetic data generation and lack contributing sufficient variation. IP bagging and costing are both exclusively based on resampling observed data. This may become problematic especially for small sample sizes or only small stratum sizes (which can occur in the resampled data sets for these two approaches): The fine structure in the given data can be spurious due to the deficit of observations. Also, due to small sample sizes and hence too few values in the sample only covering a restricted range, one may underestimate variance and covariance of the data.

In this section, we propose two procedures which aim to conquer the problem of small strata by increasing the number of observations per stratum and at the same time estimate the covariance of the population appropriately. The idea behind both approaches is to exploit the fact that within each stratum *h* all observations are assigned the same weight *w*_*h*_. This enables parametric resampling within each stratum.

Let ${\tilde{ℒ}}_{h}$ be the distribution which ${\tilde{X}}_{h}$ follows. We aim to approximate ${\tilde{ℒ}}_{h}$ by theoretical distributions and estimate their parameters for each stratum *h*. In practice, determining the multivariate distribution of the features is difficult and relies on assumptions. One might, for example, assume normally distributed features,

(5) $$\begin{array}{c} {\tilde{X}}_{h}\sim N\left(\;\mu_{h},\Sigma_{h}\;\right), \end{array}$$

and would then have to estimate ${\hat{\mu }}_{h}$ and ${\hat{\Sigma }}_{h}$ for all *h*, which is typically done by their empirical pendants. Even though we focus on the normal distribution in our empirical investigations, we propose the following approaches such that they can be applied to arbitrary distribution assumptions.

*Stochastic Inverse-Probability Oversampling.* Our first approach builds upon the re- or oversampling techniques described in Section 3.1.1. However, the repeated occurrence of observations of continuous features falsifies the covariance structure of the reconstructed samples. Hence, we add noise to those data sets obtained via IP oversampling and thus call our proceeding* stochastic IP oversampling*.

When adding this noise, we want to retain important distribution characteristics of the respective stratum. As stated above, the stratified sample contains features ${\tilde{X}}_{h}\sim {\tilde{ℒ}}_{h}$. After performing IP oversampling, the reconstructed features ${\tilde{X}}_{h}^{\prime }$ do not follow ${\tilde{ℒ}}_{h}$ anymore. We aim to adjust ${\tilde{X}}_{h}^{\prime }$ by adding noise terms ${\tilde{\varepsilon }}_{h}$ such that ${\tilde{X}}_{h}^{\prime }+{\tilde{\varepsilon }}_{h}$ approximately follows the original distribution ${\tilde{ℒ}}_{h}$ in the sense that it agrees in expectation and covariance. In the following, we derive a respective distribution ${\tilde{ℒ}}_{h}^{adj}$ for ${\tilde{\varepsilon }}_{h}$.

We seek two conditions to hold:

(6) EX~h′+ε~h=EX~h,

(7) covX~hk′+ε~hk,X~hj′+ε~hj=covX~hk,X~hj=Σh

for all *k*, *j* ∈ {1,…, *p*} denoting the index of the features. Because of (6) and since $𝔼\left({\tilde{X}}_{h}^{\prime }\right)=𝔼\left({\tilde{X}}_{h}\right)$, we obtain

(8) $$\begin{array}{c} E\left(\;{\tilde{\varepsilon }}_{h}\;\right)=0. \end{array}$$

In the Appendix (Appendix A, (B.3)), we derive the adjusted* noise covariance matrix *$\Sigma_{h}^{adj}≔cov\left({\tilde{\varepsilon }}_{h}^{\left(k\right)},{\tilde{\varepsilon }}_{h}^{\left(j\right)}\right)$, which leads to

(9) $$\begin{array}{c} \Sigma_{h}^{adj}=\frac{w_{h}-1}{w_{h}n_{h}-1}\Sigma_{h}. \end{array}$$

For instance, when assuming a multivariate normal distribution ${\tilde{X}}_{h}\sim {\tilde{ℒ}}_{h}=𝒩\left(\mu_{h},\Sigma_{h}\right)$, the noise term

(10) $$\begin{array}{c} {\tilde{\varepsilon }}_{h}\sim {\tilde{L}}_{h}^{adj}=N\left(\;0,\frac{w_{h}-1}{w_{h}n_{h}-1}\Sigma_{h}\;\right) \end{array}$$

would retain the stratum expectation and covariance (and thus in the Gaussian case the entire distribution).

In order to make a corresponding correction method more robust, we repeat the noise-adding procedure and average over the models fitted on each of those repetitions.  Algorithm 1 displays the single steps of stochastic IP oversampling.

*Parametric Inverse-Probability Bagging.* Stochastic IP oversampling above consisted of a deterministic replication of observations followed by a stochastic alteration by adding noise. Now, we propose a completely parametric approach which we call* parametric IP bagging*. As in IP bagging, we draw bootstrap samples from the original stratified data set. This time, however, we employ parametric instead of nonparametric bootstrap and set the bootstrap sample size to *n*′. As in stochastic IP oversampling, we assume a multivariate distribution underlying the original data and estimate the parameters stratum-wise. The procedure is defined by Algorithm 2.

#### 3.1.3. Properties of Correction Approaches

So far, we described seven ways to deal with sample selection bias: no correction, IP oversampling, IP bagging, costing, modified SMOTE, stochastic IP oversampling, and parametric IP bagging. This subsection compares their characteristics. They are summarized in the left part of Table 1.

*(i) Incorporation of Weights.* Except for the noncorrection approach, all correction methods incorporate weights. As mentioned in Section 3.1.1, there are cases of sample selection bias where the bias does not affect the classifier so that correction in terms of weighting is not necessary. However, as we will elaborate in this paper on two-phase case-control studies, correction is necessary in the context of complete bias.

*(ii) Correcting Covariance Structure of Learning Data.* Sample selection bias can cause a biased covariance structure in the data. Some but not all correction approaches correct for this bias: The noncorrection approach clearly uses the biased covariance structure. Also, IP oversampling does not correct for it; the replication of observations generally leads to underestimating the covariance (cf. (B.2) in the Appendix). For modified SMOTE, the resulting covariance structure depends on the magnitude of the weights *w*_*h*_ and the degree of separation of the features into distinct clusters. For instance, a stratum with large weight *w*_*h*_ will cause a large number of newly generated observations as compared to the original number of observations. The same neighbors will be selected several times such that sufficient variation of the new observations cannot be guaranteed. This may result in a similar issue as for IP oversampling described above. All other approaches aim to obtain the right covariance structure per stratum and in the entire reconstructed sample.

*(iii) Size of Reconstructed Samples.* As a well-known fact in statistical learning, the bias of a classifier increases when the learning sample size decreases. IP bagging is based on reconstructed samples of the same size *n* as the original stratified data set. Sample sizes in costing are even smaller and vary between bootstrap samples. Particularly, the small strata contain a small number of observations for these two ways of reconstructing the sample. Consequently, a certain structure of the data may get lost for learning (e.g., the appropriate variability within small strata may not be given anymore). IP oversampling, modified SMOTE, and our own methods, stochastic IP oversampling and parametric IP bagging, on the other hand, employ reconstructed samples of larger sizes *n*′ as defined in (3). By this, we intend to have sufficient numbers of observations in each stratum for possibly improving the learning of the classifier as compared to the use of smaller samples. In the nonparametric IP oversampling, the larger sample size induces a large number of perfectly repeated observations. This, again, biases the covariance structure. In our parametric approaches, stochastic IP oversampling and parametric IP bagging, this drawback does not occur.

### 3.2. Classifiers

In Sections 3.1.1 and 3.1.2, several approaches adjusting for sample selection bias have been presented and proposed. We implemented all approaches for the following classifiers: classical logistic regression based on maximum likelihood estimation as a classifier serving as reference since correction approaches are well established for it, the tree-based random forest as our main object of interest, and logistic regression including interaction terms and the naive Bayes classifier as further algorithms for comparison.

As described by Zadrozny [12], a classifiers' output can depend either on *P*(*Y*∣**x**) only or on both *P*(*Y*∣**x**) and *P*(**X**). The first type of classifiers per definition is not affected by feature bias whereas the second type is affected. Thus, one has to consider that the two types behave differently under complete bias, as well.

*Logistic Regression.* We employ logistic regression [26] as a common classical binary classification method. The model assumes *Y*∣**X** to be Bernoulli distributed with success probability

(11) $$\begin{array}{c} P\left(\;Y=1\;|\;X\;\right)={\left(\;1+\exp\left\{\;-\left(\;\beta_{0}+X\beta \;\right)\;\right\}\;\right)}^{-1}, \end{array}$$

where *β*_0_ and **β** = (*β*_1_,…,*β*_*p*_)′ are unknown parameters representing the effects of the features **X** on the outcome variable *Y*.

We investigate two variants of this model: Once, all features enter the model just linearly. In a refinement, features are additionally included as all possible two-way interaction term combinations, not only in order to detect possible interaction effects but also to obtain more complex decision boundaries.

*Random Forest.* Random forests are ensembles of decision trees and a modification of bagging [27]. The basic procedure of the learning algorithm is the following:

1. A bootstrap sample is drawn from the given learning data set.
2. A decision tree is grown by constructing recursive binary splits to the given data based on the features.
3. At each node only a subset of features is selected at random.
4. Steps (1) to (3) are repeated and all trees are averaged; class probabilities can be estimated as the relative frequency of the class of interest for a terminal node.

An essential step which is different from common bagging (cf. Section 3.1.1) is Step (3). The random selection of features decorrelates the trees and makes the bagging procedure more efficient. For all approaches in Sections 3.1.1 and 3.1.2 which are based on aggregating after resampling, namely, IP bagging, costing, stochastic IP oversampling, and parametric IP bagging, we incorporate these approaches into the random forest correspondingly. That means, instead of performing bagging within another bagging, we combine the two procedures. Note that IP oversampling incorporated in a random forest turns the approach to a bagging method. In fact, IP oversampling is exactly the same method as IP bagging when using samples of size *n*′ instead of *n*. Thus, for the implementation of our approaches into the random forest, we implicitly take both versions of IP bagging into account.

*Naive Bayes.* The naive Bayes classifier is another common machine learning algorithm for classification (see, e.g., Hastie et al. [28]). It assumes independence between the *p* features and simply calculates for each class *j* that can be attained by *Y* the marginal classifier

(12) $$\begin{array}{c} \varphi^{\left(j\right)}\left(\;X\;\right)=\overset{p}{\underset{k=1}{\prod }}\varphi^{\left(j,k\right)}\left(\;X^{\left(k\right)}\;\right) \end{array}$$

by estimating feature-wise classifiers *φ*^(*j*, *k*)^ via one-dimensional kernel-density estimation. That means the impact of each feature **X**^(*k*)^ is estimated separately and combined to an overall classifier.

## 4. Simulation Study

So far, we have presented and developed strategies for fitting classifiers under complete bias. In this section, we investigate their performance when a sample from a two-phase case-control study is given as learning data set but the test data is unbiased (i.e., it is a random sample from the population). We do this in a simulation study. After stating the setup in Section 4.1, we compare performances for the introduced correction approaches (Section 3.1) and classifiers (Section 3.2) and report the results in Section 4.2.

### 4.1. Design

For evaluating the performance of correction approaches on training samples from two-phase case-control studies and unbiased validation data sets, we need three kinds of data sets: first, a biased learning data set stemming from a two-phase case-control study; second, an unbiased large reference learning data set for comparison purposes (we refer to this data as* population*; it is not available in practice); third, an unbiased test data set distributed like the population. We artificially simulated such data sets as described in the following.

We started by generating the large unbiased population data set. To that end, we randomly sampled 10^5^ feature vectors consisting of one binary exposure variable *X*_*e*_ and *p* = 5 continuous other features ${\tilde{X}}^{\left(j\right)}$, *j* ∈ {1,…, 5}. The exposure *X*_*e*_ was meant to serve as a stratum feature with a low proportion (10%) of exposed (*X*_*e*_ = 1) individuals and a majority of nonexposed (*X*_*e*_ = 0) individuals. The *p* = 5 other features were generated independently of *x*_*e*_ and of each other. We investigated the following four distribution families:

- Normal distribution: ${\tilde{X}}^{\left(j\right)}\sim 𝒩\left(\mu^{\left(j\right)},{\sigma^{\left(j\right)}}^{2}\right)$ for all *j* = 1,…, *p*
- Student's t-distribution: ${\tilde{X}}^{\left(j\right)}\sim t(v_{j})$ for all *j* = 1,…, *p*
- Poisson distribution: ${\tilde{X}}^{\left(j\right)}\sim Po(\lambda_{j})$ for all *j* = 1,…, *p*
- Bernoulli distribution: ${\tilde{X}}^{\left(j\right)}\sim Ber(\pi_{j})$ for all *j* = 1,…, *p*

The distribution parameters were uniformly drawn from the following sets for *j* = 1,…, *p*: *μ*^(*j*)^ ∈ [1,10], *σ*^(*j*)^ ∈ [1,5], *v*_*j*_ ∈ {10,11,12,…, 98,99,100}, *λ*_*j*_ ∈ {1,2, 3,4, 5}, and *π*_*j*_ ∈ [0.4,0.6].

In order to also investigate more realistic distribution scenarios, we additionally generated and analyzed data sets with dependent features and features from different distributions. These studies yield similar results as the setting above and are described in the Supplementary Material of this paper (available online at https://doi.org/10.1155/2017/7847531).

Given the covariates $X=\left(X_{e},\tilde{X}\right)$, the outcome *Y* was generated according to a logistic regression model: *Y*∣**X** ~ Ber(*θ*(**X**)), where *θ*(**X**) = (1 + exp⁡{−(*β*_0_ + **Xβ**)})^−1^. We chose the effects in terms of regression coefficients **β** = (*β*_*e*_, *β*_1_,…,*β*_5_)′ as follows: The exposure has a negative effect on the outcome with *β*_*e*_≔log⁡0.5. The effects *β*_1_,…, *β*_5_ for the main features are varied at random, namely, uniformly on the interval [−0.15,0.15] in order to gain an intermediate performance of a classifier applied on an independent data set. *β*_0_ was chosen such that *P*(*Y* = 1) = 0.1. By this setup, the population with a rare exposure, *P*(*X*_*e*_ = 1) = 0.1, and rare cases, *P*(*Y* = 1) = 0.1, is fully generated.

In order to obtain a biased stratified sample, we simulated a two-phase random selection process from the population (Figure 1(a)) such that *P*(*Y* = 1∣*S*) = 0.5 and *P*(*X*_*e*_ = 1∣*S*) = 0.5. In a first step, an equal number of observations were randomly taken with *x*_*e*_ = 1 and with *x*_*e*_ = 0. In a second step, in each of these two strata from the first step, an equal number of observations with *y* = 1 and *y* = 0 were selected. By this, we partitioned the population into four equally sized strata corresponding to (*y*, *x*_*e*_) ∈ {(1,1), (1,0), (0,1), (0,0)}.

Test data sets of size 10^4^ were created in exactly the same way as the population. For our simulation study, we generated the population data set, the stratified data set, and the test set 1000 times for each feature distribution assumption. This way, we could empirically assess the variability of the performance of the correction and classification methods.

*Application of Classifiers.* We apply the seven correction approaches (Section 3.1) combined with the four considered classifiers (Section 3.2) to the synthetic data. To that end, stochastic IP oversampling and parametric IP bagging, proposed by us (Section 3.1.2), require a distribution assumption for the main features $\tilde{X}$. We always assume them to be normally distributed, even if the features in fact follow a Student's t-, Poisson, or Bernoulli distribution. We aim to find out how the algorithms get affected when assumptions are not met.

In fact, the four different distribution scenarios meet the Gaussian assumption in decreasing order: The normal distribution trivially fulfills it. The t-distribution is still continuous and symmetric so that the violation of the normality assumption may not get too severe. The Poisson distribution is discrete but approximately normal for *λ* ≥ 30; however, in order to guarantee the normality assumption to be violated, we let *λ*_*i*_ ∈ {1,2, 3,4, 5}. The Bernoulli distribution cannot be seen as continuous and violates the normality assumption the most.

*Evaluation.* We measure the performance of the different classifiers combined with the various correction approaches by the Area-under-the-Receiver-Operating-Characteristic curve (AUC) [29]. The AUC is appropriate especially in the context of sample selection bias since it does not require binary prediction (i.e., discretizing continuous risks by choosing a cut-off) and is unaffected by linear transformations of the predictions as only ranks are considered. Thus, differences in performance should not be influenced by good or bad calibration of the prediction.

The goal of the comparison is to see whether correction approaches perform significantly better than not correcting. For each classifier, we fit a linear regression model with the AUC as target variable and the correction approach as covariate. The latter variable is dummy-coded with “no correction” as reference category. An approach is determined to differ significantly from the noncorrection approach if its coefficient's *t*-test confidence interval does not contain zero. For all comparisons, we use a level of significance of *α* = 5%.

*Software.* We used the statistical software R for all analyses [30]. More specifically, for building logistic regression models, we used the R package* stats* [30], for random forest the R package* ranger* [31], and for naive Bayes the R package* e1071* [32]. The modified implementation of the SMOTE algorithm is based on the R package* smotefamily* [33]. We validated our results via ROC analysis, using the R packages* pROC* [34] and* ROCR* [35].

### 4.2. Results

**Figure 3:**
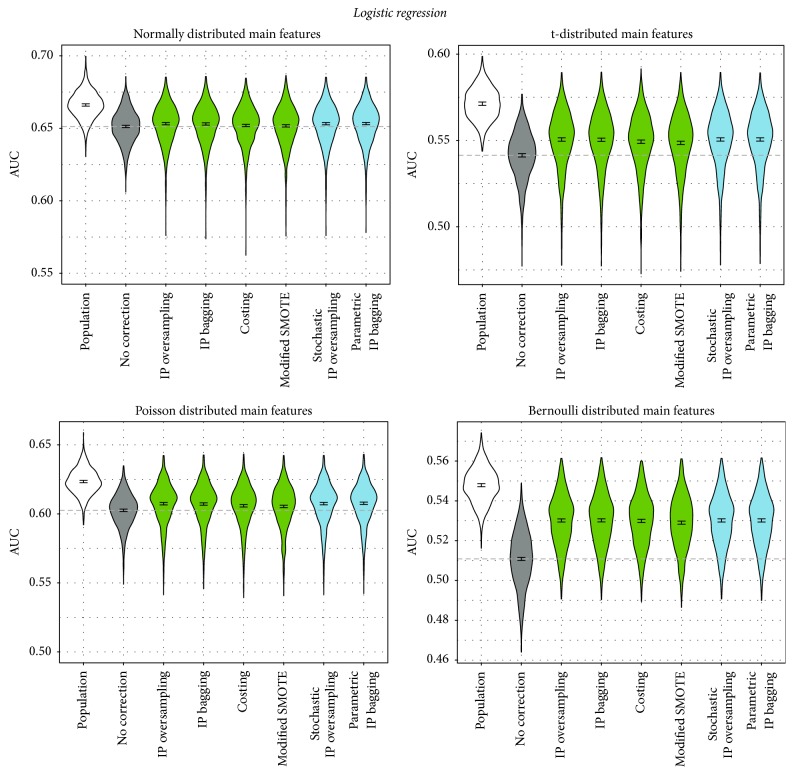
Performance of correction approaches in logistic regression, measured by AUC. We fit a linear model for the AUC as influenced by the correction method (dummy-coded, no correction as reference category). The graphic depicts 95% confidence intervals for the respective coefficients. The dashed line shows the intercept of the model (i.e., the mean AUC for no correction). The blue colored methods are newly proposed in this paper.

**Figure 4:**
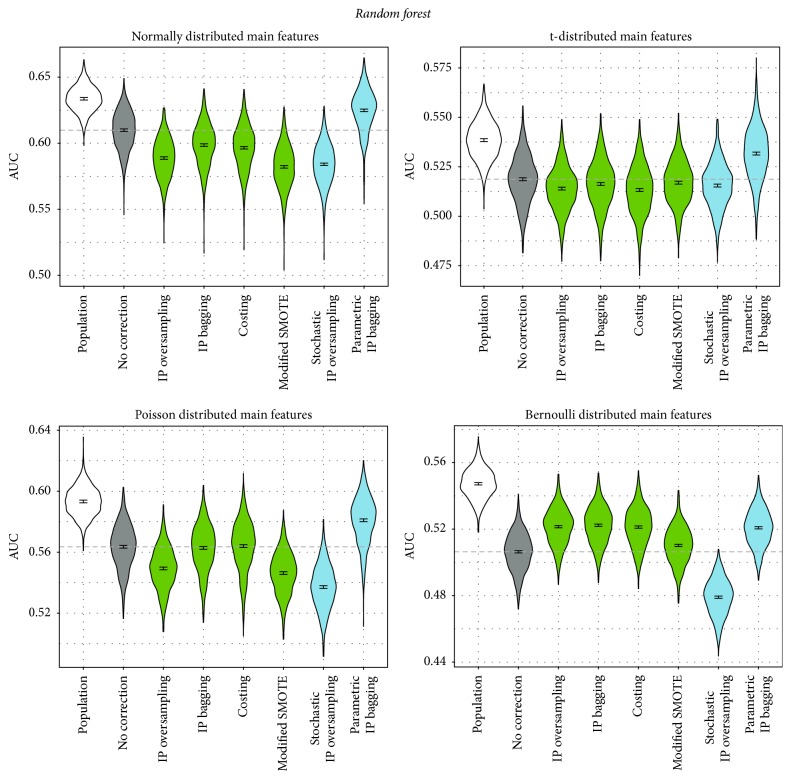
Performance of correction approaches in the random forest, measured by AUC. We fit a linear model for the AUC as influenced by the correction method (dummy-coded, no correction as reference category). The graphic depicts 95% confidence intervals for the respective coefficients. The dashed line shows the intercept of the model (i.e., the mean AUC for no correction). The blue colored methods are newly proposed in this paper.

**Figure 5:**
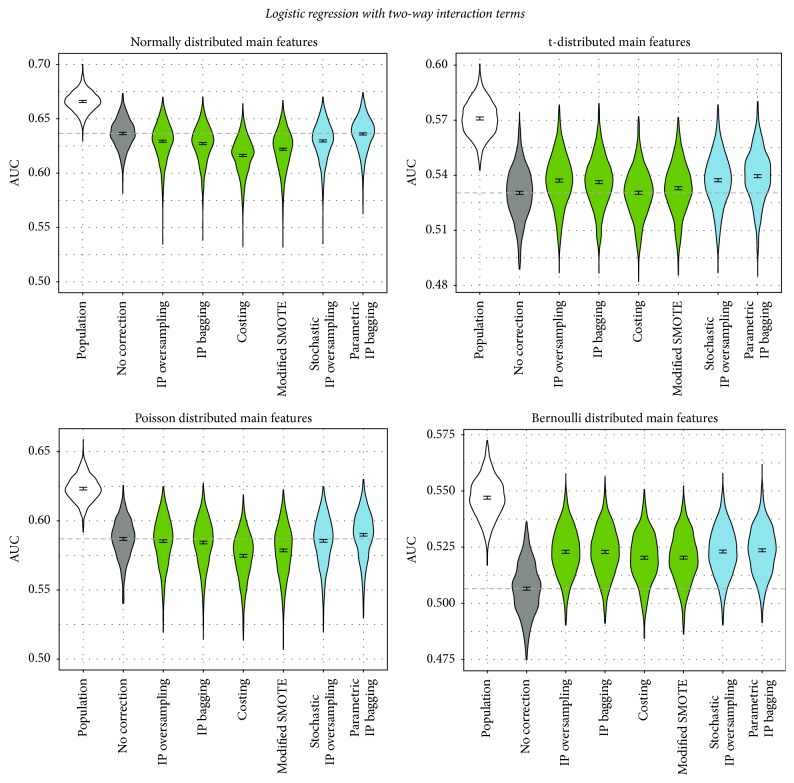
Performance of correction approaches in logistic regression with additional two-way interaction terms, measured by AUC. We fit a linear model for the AUC as influenced by the correction method (dummy-coded, no correction as reference category). The graphic depicts 95% confidence intervals for the respective coefficients. The dashed line shows the intercept of the model (i.e., the mean AUC for no correction). The blue colored methods are newly proposed in this paper.

**Figure 6:**
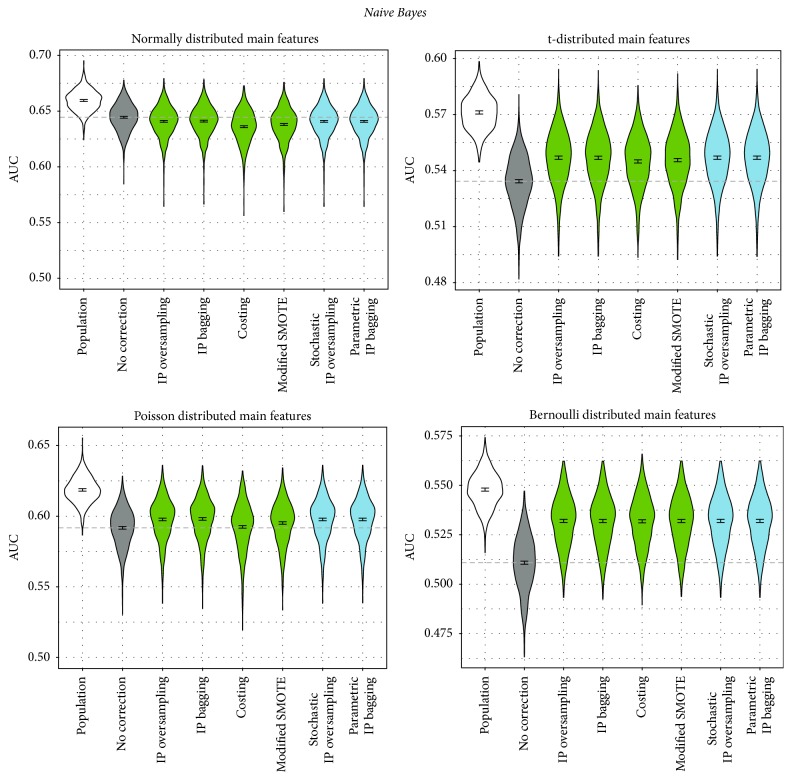
Performance of correction approaches in the naive Bayes classifier, measured by AUC. We fit a linear model for the AUC as influenced by the correction method (dummy-coded, no correction as reference category). The graphic depicts 95% confidence intervals for the respective coefficients. The dashed line shows the intercept of the model (i.e., the mean AUC for no correction). The blue colored methods are newly proposed in this paper.

The simulation study yielded the following results (see also Figures 3–6): As expected, for every distribution scenario (see previous subsection) and all classifiers, the performance of learning on the entire population was significantly better than learning without correction on the smaller biased learning data set. Also, for all classifiers and in all distribution scenarios, there was at least one correction technique that outperformed the noncorrection approach (with two exceptions: logistic regression with additional interaction terms and naive Bayes, both in case of normally distributed main features).

However, there were differences between classifiers concerning the success of correction approaches. We start by contrasting logistic regression and the random forest as this comparison is of our primary interest.

The overall result for logistic regression (Figure 3) is that all correction approaches perform significantly better than noncorrection. Exceptions are costing and modified SMOTE in the normal distribution scenario which on average performs better than noncorrecting, but not significantly. For t-distributed and Poisson distributed features, the difference between the performance of noncorrection and the other approaches is more prominent than for the normal distribution scenario. In the Bernoulli case, this difference is the highest. Within each distribution scenario, the correction approaches perform similarly to each other.

For the random forest, the picture is rather different (Figure 4): Only one correction approach performs significantly better than noncorrecting: the parametric IP bagging proposed in this paper. In fact, for normally and t-distributed features, all other correction methods perform even worse than noncorrecting. In the Poisson scenario, they perform either worse than noncorrection or equally fine (IP bagging and costing). Only in the scenario in which the assumption of having continuous main features (required by the approaches proposed by us) is not met at all (i.e., for the Bernoulli distribution) do almost all correction approaches perform better than not correcting. An exception is stochastic IP oversampling proposed by us. This approach failed in all distribution scenarios for the random forest.

Table 1 summarizes the properties of the correction approaches (Section 3.1.3) together with the just described results. We label the performance of an approach to be sufficient if it results in a significant increase of the AUC as compared to the noncorrection approach for the normal distribution scenario. Costing and modified SMOTE do not yield unambiguous improvements for logistic regression since their confidence intervals slightly overlap with the value under the null hypothesis. However, as we will see in Section 5, both approaches perform significantly better than noncorrection on real data.

In order to obtain a more comprehensive picture of the benefit of correcting for sample selection bias, we applied the correction methods in combination with two more classifiers, logistic regression with additional two-way interaction terms in addition to the linear terms and naive Bayes, leading to the following results.

Logistic regression with interaction terms yields a similar picture as standard logistic regression (Figure 5): All correction approaches perform similarly to each other. In the t- and Bernoulli scenario, again all correction approaches outperform the noncorrection approach, except for costing for t-distributed features, which performs similarly to noncorrecting. For both the normal and the Poisson distribution, all correction approaches perform significantly worse than not correcting. An exception is parametric IP bagging: Similar to the random forest case, only this method performs significantly better than no correction for the Poisson distribution scenario. For the normal distribution, the approach is the only one which does not perform significantly worse than the noncorrecting approach.

For naive Bayes (Figure 6), again all correction approaches behave similarly as in logistic regression. Depending on the data distribution, correction approaches perform worse or better than noncorrection. Especially in the normal distribution scenario, the correction approaches are not successful.

## 5. Real Data Application

This section investigates the performance of the correction methods in a real data example. Other than in the synthetic data situation in the previous section, we do not know the true distribution of the entire population here. In order to still be able to evaluate the predictions appropriately, we chose a very large real data set from which we could extract a small stratified learning set and a large unbiased test set as described in the following.

### 5.1. Design

**Figure 7:**
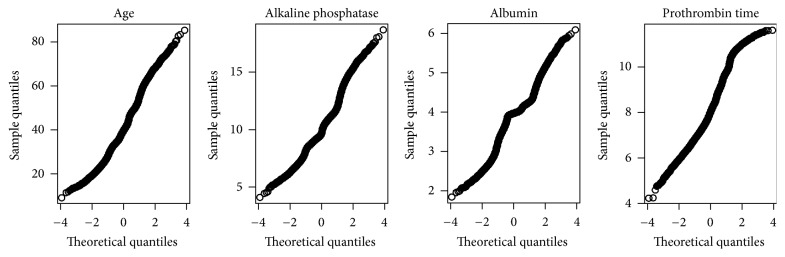
Normal quantile-quantile plots for main features $\tilde{X}$ in real data set. For visualization purposes, we only displayed a random sample of 10,000 observations instead of the full data set of size 10^6^.

*Data.* We evaluate the various prediction methods on the example of the* hepatitis* data set (data ID: 269, exact name: “BNG (hepatitis),” version: 1) from OpenML [36]. It contains 10^6^ observations of a binary outcome *Y* and 20 features. *Y* captures whether a hepatitis patient stayed alive and hence takes the categories* live* and* die*. We chose the binary variable* sex* as stratum feature *X*_*e*_. From the remaining variables, we took into account the four continuous features* albumin*,* alkaline phosphatase*,* prothrombin time*, and* age*, denoted by $\tilde{X}$. These features were approximately normally distributed (partly after transformation; see the quantile-quantile plots in Figure 7) and strongly associated with the outcome.

**Figure 8:**
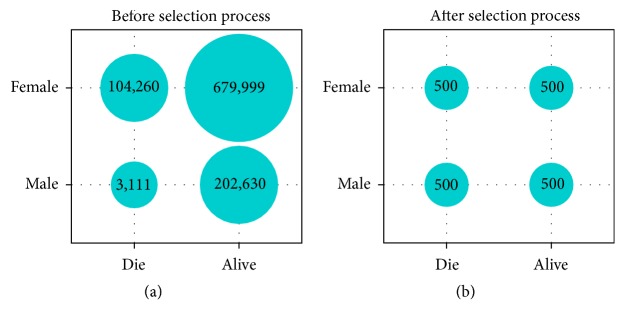
Cross table for the* hepatitis* data set before (a) and after (b) the selection process of a two-phase case-control study.

*Stratification Process.* We aimed to evaluate the prediction methods on data sets which underwent sample selection bias. We hence constructed a learning data set by performing a two-phase stratified random selection process on the* hepatitis* data set. To that end, we selected *n* = 2000 out of the 10^6^ observations, enriching the outcome *Y* and the feature variable* sex*, denoted by *X*_*e*_.  Figure 8 shows the sizes of the four strata in analogy to Figure 1(b). As test data set, we chose a subset of 10,000 observations from the hepatitis data set, disjoint to the learning data. We defined the first 10^6^ observations (without the test data) as the population which served as reference learning data set as in the previous section.

### 5.2. Results

We trained all methods on the biased learning data and evaluated them on the unbiased test data. The resulting AUCs are compared by seven pairwise hypothesis tests according to [37]. We corrected for multiple testing via Bonferroni correction (i.e., set the threshold for *p* values to *α*^*∗*^ = 0.05/7 = 0.0071).

**Figure 9:**
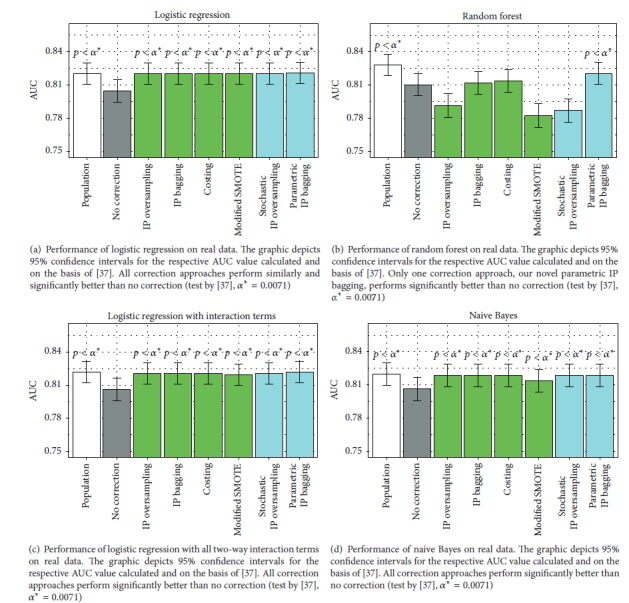


The real data results confirm the findings from the simulation study. For logistic regression, all weighting approaches perform very similarly, which was significantly better than the nonweighting approach and even comparable to learning on a large population (Figure 9(a)).

For random forest, we obtain similar results as in the simulation study (Figure 9(b)): Only parametric IP bagging performs significantly better than the nonweighting approach. Costing and IP bagging perform insignificantly better; IP oversampling, modified SMOTE, and stochastic IP oversampling perform significantly worse.

Also, for logistic regression with interaction terms and naive Bayes, we obtain results matching with the simulation study: The assumptions for normality are met only roughly for the real data, in which case the correction approaches all perform similarly and better than no correction (Figure 9(d)).

## 6. Discussion and Conclusion

We investigated how to learn classifiers on stratified random samples as resulting from two-phase case-control studies. Here, our emphasis was on random forest classification since previous bias correction methods did not pay special attention to resampling-based classifiers. However, we studied a broad range of classification techniques. This work hence guides the choice of such approaches also for other classifiers. The methods are immediately applicable due to the implementations provided in our R package* sambia*.

Both our simulation study and the real data application show that for classifiers trained on biased data sets prediction on unbiased data sets can be improved if the stratification process is taken into account and corrected for. However, state-of-the-art correction approaches from classical statistics (IP oversampling, IP bagging, costing, and modified SMOTE) do not yield the desired improvement for random forests. In fact, they can even lead to worse AUC values than those obtained when not performing any correction. From our two proposed approaches (stochastic IP oversampling and parametric IP bagging), on the other hand, the latter could always outperform the noncorrection approach.

We were also interested in all correction approaches' success when employed in the context of logistic regression. It turned out that any method improves prediction on an independent data set as compared to no correction, and all correction techniques perform similarly.

Table 1 helps to explain the different behaviors of the two classifiers: Correction approaches are based on one or several of the principles (i) IP weighting, (ii) rebuilding the original covariance structure, and (iii) increasing the number of learning observations as compared to the stratified sample. Obviously, weighting (Property (i)) should be applied in order to obtain any improvement in performance. Moreover, the covariance structure should be corrected for (Property (ii)) when applying a random forest. IP oversampling and partly modified SMOTE failed to fulfill this criterion. For logistic regression, in contrast, the covariance structure does not matter since point estimates of regression coefficients are not affected when the variance in the data is underestimated. Last, sample sizes (Property (iii)) seem to matter more for random forests than for logistic regression. This is reasonable since too small sample sizes can restrict the range of the values of a feature and thus underestimate their variance leading to the same issue as for Property (ii). This made IP bagging and costing perform poorly for the random forest. This leaves us with stochastic IP oversampling and parametric IP bagging, both proposed in this paper. However, although stochastic IP oversampling was designed to fulfill Properties (i), (ii), and (iii), we could not yield successful results for random forests.

Having compared correction methods in random forests and in logistic regression, one may conclude that the choice of parametric IP bagging is advisable whenever the distribution assumptions for this approach are met. In order to once more revise this conclusion, we investigated the behaviors of all correction approaches in two more classifiers, a logistic regression model with additional interaction terms and the naive Bayes classifier. For the logistic regression model with interaction terms, once again only the parametric IP bagging consistently outperformed the noncorrection approach. For naive Bayes, all approaches performed similarly among each other, confirming the above stated rule.

Against our expectations, naive Bayes failed in the simulation study for the normal distribution scenario but did well for all other distributions. A generally unexpected result was the poor accomplishment of stochastic IP oversampling. It performed worse than noncorrection in several scenarios and was successful only in those situations where all other correction approaches were successful as well.

For a random forest, parametric IP bagging is an effective technique for prediction on an unbiased data set and can also be preferred for other classifiers. However, in this paper, we restricted our simulations and real data example to the case where the main features could be assumed to be roughly normally distributed (after transformation, if necessary) so that the assumption of a multivariate normal distribution was appropriate. The success of parametric IP bagging generally depends on meeting the assumptions about the distributions of the features. Hence, the method should be chosen with care. On the other hand, our simulations show that, even in scenarios where assumptions are barely met (e.g., for Poisson distributed features), the approach still works. Clearly, one could also adjust the distribution family for the parametric bootstrap in parametric IP bagging. Even mixture distributions are conceivable (e.g., for bimodal feature distributions).

So far, parametric IP bagging has not been designed for binary or categorical main features or combinations of different types. This could be done by subgrouping the corresponding categories (or combining categories in the case of several categorical features) and estimating parameters in each of the subgroups for the assumed distribution family analogously to what we did for the different strata. Again, one would draw parametric bootstrap samples within all subgroups and construct a new unbiased sample within the scope of parametric IP bagging.

Even though our new approaches were developed for the random forest, they are generally tailored towards learning by any classifier and can be incorporated in other machine learning algorithms. Parametric IP bagging has been shown to perform well even if theoretical assumptions are not met. It can be applied on any stratified random sample and is not restricted to two-phase case-control studies. More generally, it is suited for any sample suffering from sample selection bias where the stratum features are categorical and the remaining features roughly follow a multivariate distribution from which parametric bootstrap samples can be drawn. For general classifiers, its performance is mostly comparable to that of other correction methods. Parametric IP bagging is the first correction method designed for the random forest and in that context clearly outperforms all other approaches.

## Supplementary Material

The file contains results for an additional simulation setting with predictor variables from different distribution families.

## Figures and Tables

**Algorithm 1:**
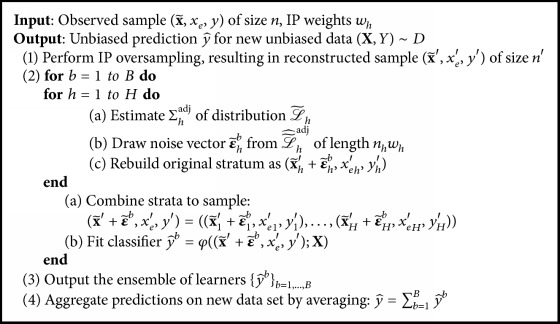
Stochastic inverse-probability oversampling.

**Algorithm 2:**
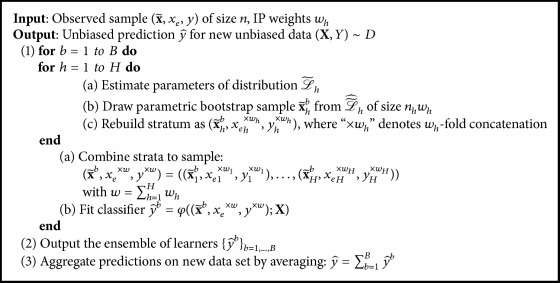
Parametric inverse-probability bagging.

**Table 1:** Properties and performance of correction approaches for logistic regression and random forest. The properties are as follows: (i) a correction attempt is made at all; (ii) the covariance structure of the learning data is attempted to be unbiased; (iii) learning is based on a data set containing a larger number *n*′ of observations than the original stratified data set (see (3)). Criteria are fulfilled (“✓”), not clearly fulfilled (“(✓)”), or not fulfilled (“×”).

Correction approach	Properties according to Section 3.1.3			Sufficient performance	
	(i)	(ii)	(iii)	Logistic regression	Random forest
No correction	×	×	×	×	×
IP oversampling	✓	×	✓	✓	×
IP bagging	✓	✓	×	✓	×
Costing	✓	✓	×	(✓)	×
Modified SMOTE	✓	(✓)	✓	(✓)	×
Stochastic IP oversampling	✓	✓	✓	✓	×
Parametric IP bagging	✓	✓	✓	✓	✓

## Appendix

### A. Dependence of *S* on X and *Y* for Label and Feature Bias

Label bias does not imply that *S* is independent of **X**; that is,

(A.1) PS ∣ X,Y=PS ∣ Y∧PS ∣ Y≠PS⇏PS ∣ X=PS.

Proof Let **x**≔*t*(*y*), where *t* is a function mapping to {0,1}. Then, *P*(*S*∣**x**) = *P*(*S*∣*t*(*y*)) = *P*(*S*∣*y*) ≠ *P*(*S*).

Analogously, one can show that feature bias does not imply that *S* is independent of *Y*.

### B. Covariance Matrix of Noise in Stochastic IP Oversampling

Here, we derive an appropriate noise covariance matrix to be added to the features ${\tilde{X}}_{h}^{\prime }$ resulting from IP oversampling.

For one stratum *h*, we look at the covariance of the pair of features ${\tilde{X}}_{h}^{\left(k\right)}$, ${\tilde{X}}_{h}^{\left(j\right)}$ for *k*, *j* ∈ {1,…, *p*}. For sample size *n*, we get per stratum a sample covariance per pair ${\tilde{x}}_{h}^{\left(k\right)}$, ${\tilde{x}}_{h}^{\left(j\right)}$, given by

(B.1) $$\begin{array}{c} s_{{\tilde{x}}_{h}^{\left(k\right)},{\tilde{x}}_{h}^{\left(j\right)}}=\frac{1}{n_{h}-1}\overset{n_{h}}{\underset{i=1}{\sum }}\left(\;{\tilde{x}}_{hi}^{\left(k\right)}-{\overset{-}{x}}_{h}^{\left(k\right)}\;\right)\left(\;{\tilde{x}}_{hi}^{\left(j\right)}-{\overset{-}{x}}_{h}^{\left(j\right)}\;\right), \end{array}$$

where ${\overset{-}{x}}_{h}^{\left(l\right)}≔\left(1/n_{h}\right)\sum_{i=1}^{n_{h}}{\tilde{x}}_{hi}^{\left(l\right)}$ for any *l* ∈ {1,…, *p*}.

For IP oversampling, we replicate the data points by the factor *w*_*h*_, which varies per stratum. Thus, the covariance of the modified sample is

(B.2) sx~hk,x~hj′=1whnh−1∑i=1nhwhx~hik−x−hkx~hij−x−hj=whnh−1whnh−1sx~hk,x~hj.

In addition to simple IP oversampling, stochastic IP oversampling incorporates the summation of some noise (matrix) $\tilde{\varepsilon }$. We want the following to hold for a pair of the random vectors ${\tilde{\varepsilon }}^{\left(k\right)}$, ${\tilde{\varepsilon }}^{\left(j\right)}$ of size *n*_*h*_:

(B.3) $$\begin{array}{c} cov\left(\;{\tilde{X}}_{h}^{\left(k\right)\prime }+{\tilde{\varepsilon }}_{h}^{\left(k\right)},{\tilde{X}}_{h}^{\left(j\right)\prime }+{\tilde{\varepsilon }}_{h}^{\left(j\right)}\;\right)=cov\left(\;{\tilde{X}}_{h}^{\left(k\right)},{\tilde{X}}_{h}^{\left(j\right)}\;\right), \end{array}$$

where ${\tilde{X}}_{h}^{(k)\prime }$, ${\tilde{X}}_{h}^{(j)\prime }$ are the random variables resulting from replication by a factor *w*_*h*_ (oversampling).

We can simplify

(B.4) covX~hk′+ε~hk,X~hj′+ε~hj=covX~hk′,X~hj′+covX~hk′,ε~hj+covX~hj′,ε~hk+covε~hk,ε~hj=covX~hk′,X~hj′+covε~hk,ε~hj,

since the noise component ${\tilde{\varepsilon }}_{h}^{\left(j\right)}$ should not correlate with the feature random vector **X**_*k*_ (neither ${\tilde{\varepsilon }}_{h}^{\left(k\right)}$ with ${\tilde{X}}_{h}^{\left(j\right)}$, resp.). This also holds for *j* = *k*.

We can estimate the components of the covariance matrix $cov\left({\tilde{X}}_{h}^{\left(k\right)\prime },{\tilde{X}}_{h}^{\left(j\right)\prime }\right)$ by $s_{{\tilde{x}}_{h}^{\left(k\right)},{\tilde{x}}_{h}^{\left(j\right)}}^{\prime }=\left(w_{h}(n_{h}-1)/\left(w_{h}n_{h}-1\right)\right)s_{{\tilde{x}}_{h}^{\left(k\right)},{\tilde{x}}_{h}^{\left(j\right)}}$. Substituting this into (B.3) yields, for the entries of our* noise covariance matrix*,

(B.5) sε~hk,ε~hj′=sx~hk,x~hj−whnh−1whnh−1sx~hk,x~hj=wh−1whnh−1sx~hk,x~hj.

In terms of random variables, the empirical covariance matrix combining all entries $s_{{\tilde{\varepsilon }}_{h}^{\left(k\right)},{\tilde{\varepsilon }}_{h}^{\left(j\right)}}^{\prime }$ for all *k*, *j* ∈ {1,…, *p*} would be replaced by Σ_*h*_^adj^ and the empirical covariance matrix combining all entries $s_{{\tilde{x}}_{h}^{\left(k\right)},{\tilde{x}}_{h}^{\left(j\right)}}$ for all *k*, *j* ∈ {1,…, *p*} by Σ_*h*_.

## Additional Points

*Supplementary Material.* Additional figures, code, and data are available at https://www.helmholtz-muenchen.de/index.php?id=47085.
