# Supplementary material for: Correcting Classifiers for Sample Selection Bias in Two-Phase Case-Control Studies
Source: Comput Math Methods Med. 2017 Sep 24;2017:7847531. doi: 10.1155/2017/7847531 (PMC5632994; doi:10.1155/2017/7847531)
Supplement: Supplementary file 1 — The file contains results for an additional simulation setting with predictor variables from different distribution families. [file 7847531.f1.pdf]

Supplementary Material for 'Correcting classifiers for sample selection bias in two-phase case-control studies'

Norbert Krautenbacher[1,2]

Fabian J. Theis[1,2]

Christiane Fuchs[1,2]

September 16, 2017

1 Further simulation scenarios

In addition to Section 4 of the main paper [1] a further simulation scenario was conducted containing variables from different distributions which are partly correlated and with an interaction effect on the outcome. We also added an additional noise variable (which was not known/included for the training process.)

1.1 Design

Concretely we generated the data analogously to the other scenarios of Section 4.1 with the following changes.
The variables were generated as follows:

- $\tilde{X}^{(1)} \sim \mathcal{N}(0, 1)$
- $\tilde{X}^{(2)} \sim \mathrm{t}(25)$
- $\tilde{X}^{(3)} \sim \tilde{X}^{(1)} + \mathcal{N}(0, 0.36)$
- $\tilde{X}^{(4)} \sim \tilde{X}^{(2)} + \mathcal{N}(0, 1.69)$
- $\tilde{X}^{(5)} \sim \mathrm{Ber}(0.6)$

1 Institute of Computational Biology, Helmholtz Zentrum München, German Research Center for Environmental Health, Munich, Germany
2 Department of Mathematics, Technische Universität München, Munich, Germany
Correspondence should be addressed to Christiane Fuchs

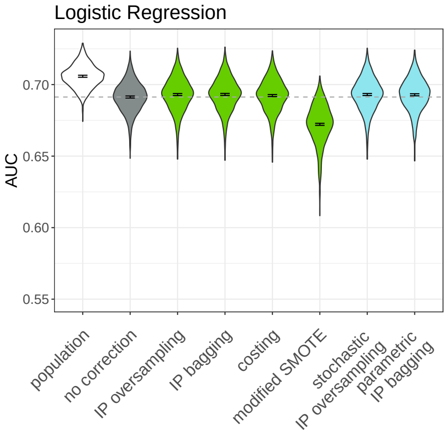

Figure 1a: Performance of correction approaches for mixed distributed features for logistic regression, measured by AUC. All approaches perform significantly better than no correction except for the modified SMOTE approach.

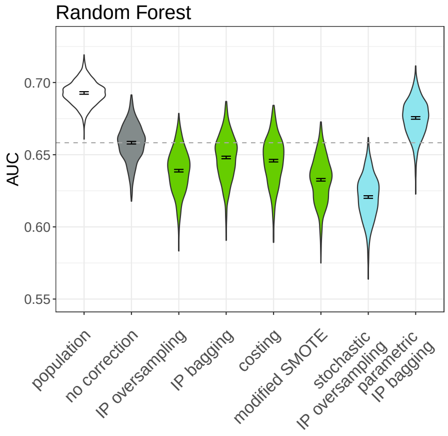

Figure 1b: Performance of correction approaches for mixed distributed features for random forest, measured by AUC. Only parametric IP bagging performs significantly better than no correction.

- $\tilde{X}^{(6)} \sim \mathcal{N}(0,1)$
- $\tilde{X}^{(7)} = \tilde{X}^{(1)} * \tilde{X}^{(5)}$

Into our models we included $\tilde{X}^{(j)}$ for $j = 1, \ldots, 5$, so that $\tilde{X}^{(6)}$ represents noise for constructing Y and $\tilde{X}^{(7)}$ an interaction. The corresponding effects were chosen to be $\boldsymbol{\beta} = (\beta_e, \beta_1, \ldots, \beta_7) = (0.5, 0.1, -0.12, 0.07, 0.05, -0.9, 0.07, 0.9)$.

1.2 Results

The performances for the simulation scenario for the four classifiers, logistic regression, random forest, logistic regression with interaction terms, and naive Bayes, are compared in Figure 1: We fit a linear model for the AUC as influenced by the correction method (dummy-coded, no correction as reference category). The graphic depicts 95% confidence intervals for the respective coefficients. The dotted line shows the intercept of the model, i.e. the mean AUC for no correction.The blue coloured methods are newly proposed in this paper.

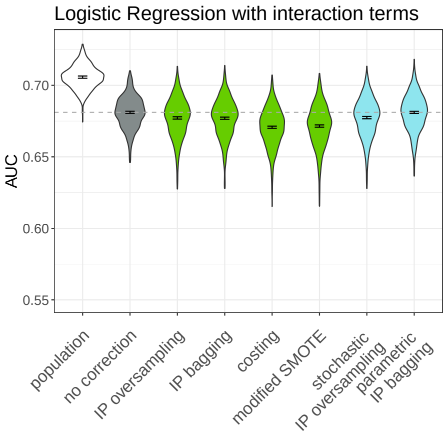

Figure 1c: Performance of correction approaches for mixed distributed features for logistic regression with interaction effects, measured by AUC. All approaches perform significantly worse than no correction except parametric IP bagging which is not significantly different.

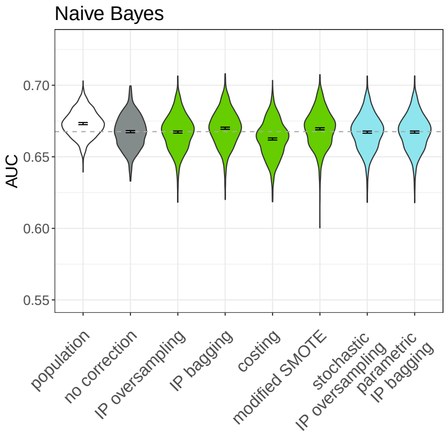

Figure 1d: Performance of correction approaches for mixed distributed features for naive Bayes, measured by AUC. Only IP bagging and modified SMOTE perform significantly better than no correction.

References

[1] Krautenbacher, N., F. J. Theis, and C. Fuchs (2017). Correcting classifiers for sample selection bias in two-phase case-control studies. *Computational and Mathematical Methods in Medicine*.
